# Human Aquaporins: Functional Diversity and Potential Roles in Infectious and Non-infectious Diseases

**DOI:** 10.3389/fgene.2021.654865

**Published:** 2021-03-16

**Authors:** Abul Kalam Azad, Topu Raihan, Jahed Ahmed, Al Hakim, Tanvir Hossain Emon, Parveen Afroz Chowdhury

**Affiliations:** ^1^Department of Genetic Engineering and Biotechnology, School of Life Sciences, Shahjalal University of Science and Technology, Sylhet, Bangladesh; ^2^Louvain Institute of Biomolecular Science and Technology, Université catholique de Louvain, Louvain-la-Neuve, Belgium; ^3^Department of Dermatology, Sylhet Women’s Medical College, Sylhet, Bangladesh

**Keywords:** human aquaporins, aquaporins and infectious diseases, water homeostasis, functional regulation, drug targets

## Abstract

Aquaporins (AQPs) are integral membrane proteins and found in all living organisms from bacteria to human. AQPs mainly involved in the transmembrane diffusion of water as well as various small solutes in a bidirectional manner are widely distributed in various human tissues. Human contains 13 AQPs (AQP0–AQP12) which are divided into three sub-classes namely orthodox aquaporin (AQP0, 1, 2, 4, 5, 6, and 8), aquaglyceroporin (AQP3, 7, 9, and 10) and super or unorthodox aquaporin (AQP11 and 12) based on their pore selectivity. Human AQPs are functionally diverse, which are involved in wide variety of non-infectious diseases including cancer, renal dysfunction, neurological disorder, epilepsy, skin disease, metabolic syndrome, and even cardiac diseases. However, the association of AQPs with infectious diseases has not been fully evaluated. Several studies have unveiled that AQPs can be regulated by microbial and parasitic infections that suggest their involvement in microbial pathogenesis, inflammation-associated responses and AQP-mediated cell water homeostasis. This review mainly aims to shed light on the involvement of AQPs in infectious and non-infectious diseases and potential AQPs-target modulators. Furthermore, AQP structures, tissue-specific distributions and their physiological relevance, functional diversity and regulations have been discussed. Altogether, this review would be useful for further investigation of AQPs as a potential therapeutic target for treatment of infectious as well as non-infectious diseases.

## Introduction

Aquaporins (AQPs) are channel-forming integral membrane proteins and found in all living organisms from bacteria to human (Agre et al., 1998; Azad et al., 2011b, 2016, 2018, 2021) and even in chlorella virus (Gazzarrini et al., 2006). AQPs mainly facilitate the transmembrane diffusion of water as well as various small solutes and are involved in cellular trafficking and many physiological processes (Hedfalk et al., 2006; Törnroth-Horsefield et al., 2006; Azad et al., 2008; Törnroth-Horsefield et al., 2010; Janosi and Ceccarelli, 2013; Kaptan et al., 2015; Pelagalli et al., 2016; Canessa Fortuna et al., 2019). Functionally diverged human AQPs are involved in wide variety of non-infectious diseases including cancer, renal dysfunction, neurological disorder, epilepsy, skin disease, metabolic syndrome, and even cardiac diseases (Verkman et al., 2008a,b, 2014; Verkerk et al., 2019). Growing data suggest their possible involvement in cell volume regulating events associated with various non-infectious diseases (Verkman et al., 2014; Pelagalli et al., 2016; Meli et al., 2018). Consequently, AQPs have become a potential drug target in clinical medicine (Verkman, 2012; Verkman et al., 2014; Esteva-Font et al., 2016; Méndez-Giménez et al., 2018; Abir-Awan et al., 2019).

**FIGURE 1:**
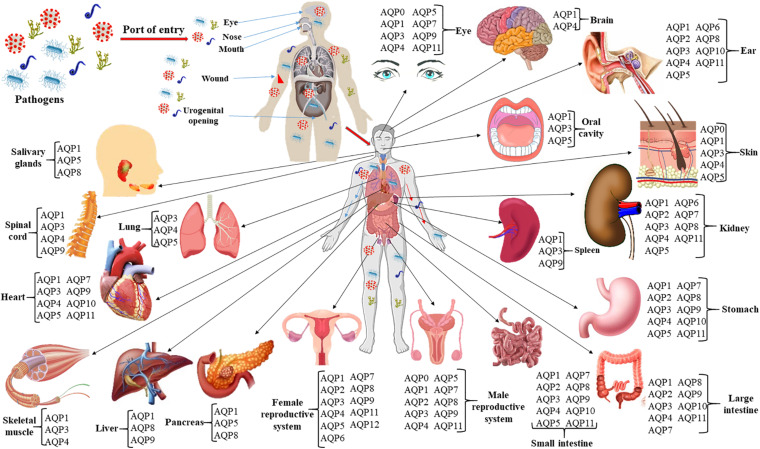
Infection route, spreading and shedding at different organs of the body and tissue-specific distribution of AQPs. The roles of AQPs in non-infectious diseases such as different cancers and tumors, cerebral edema and ischemic stroke, obesity, renal and skin diseases, and cataracts have been widely studied. At least 10 AQPs of different organs are associated with bacterial, viral, and parasitic infections. Expressions of AQPs in infectious diseases are summarized in Table 2 of the current review.

Many studies and several reviews have been done on the involvement of AQPs and their regulations in non-infectious diseases. However, limited studies have been done involving AQPs in infectious diseases although studies on plant-pathogens interactions involving AQPs have been advanced (Wang R. et al., 2018; Zhang et al., 2019; Li et al., 2020; Xu and Zwiazek, 2020). An infectious disease, an illness caused by bacteria, virus, fungi or parasite, is one of the foremost reasons of morbidity and mortality in individuals worldwide. The pathogen can spread and shed in different parts of the body as shown in Figure 1 and develop systematic or localized infection. Furthermore, dysbiosis of human microbiota imparts significant metabolic and immunologic perturbations on the host leading to many local and systemic diseases (Liang et al., 2018). Both acute and chronic inflammatory processes induced by microbial infections involve disbursement of huge metabolic energy, loss of function, tissue damage and destruction, vascular leakage and hemorrhages (Akıncı et al., 2013; Molinas et al., 2016; Meli et al., 2018). Infection-induced cell migration, production, and accumulation of effector molecules to the infected sites change the cell morphology, volume and movement, which are associated with alteration of cellular and tissue homeostasis (Medzhitov, 2008). The inability of cells to regulate fluid movement through the biological membranes results in imbalanced homeostasis and leads to severe alteration of cell physiology (Meli et al., 2018). Cells regulate their shapes and volume, and control homeostasis by utilizing water, the most abundant molecule in the body. Therefore, AQPs might have crucial roles for controlling cellular volume and homeostasis in infectious diseases.

Based on the recent studies so far done on the involvement of AQPs in infections, we herein discuss the potential roles of AQPs in infectious diseases, infection-induced inflammatory process, and in maintenance of infection-mediated loss of cellular homeostasis or tissue damage. Furthermore, the structural and functional diversity of human AQPs and their roles in non-infectious diseases, and finally AQPs as the potential drug targets are also discussed. Discussions on the roles of AQPs in infectious and non-infectious diseases in the same review will stimulate researchers to focus and explore AQPs in infectious diseases. To the best of our knowledge, it is the first review to focus on the involvement of AQPs in infectious diseases.

## Structural Properties of Human Aquaporins

**FIGURE 2:**
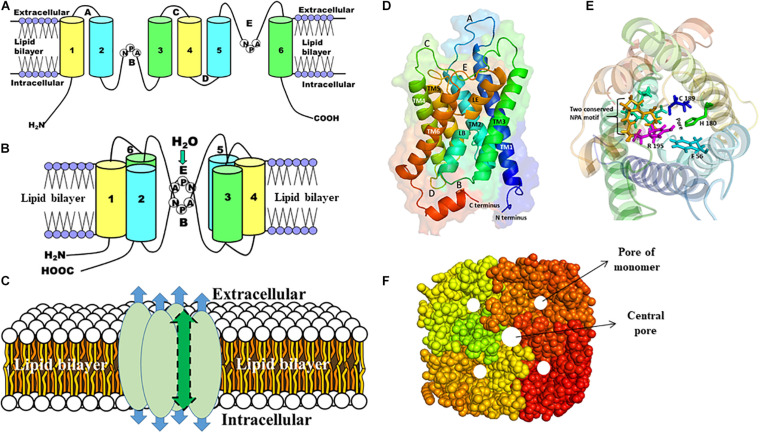
The structure of AQP monomer and homotetramers. A schematic representation of the general structures of AQP is shown **(A–C)**. **(A)** Each AQP monomer has six transmembrane domains (1–6) spanning the plasma membrane, which are connected with five loops (A–E). **(B)** Two conserved NPA motifs in loops B and E are juxtaposed oppositely to form the channel through which molecules are passed. **(C)** Each AQP monomer contains independent pore (shown as blue arrow) and the monomers are assembled as tetramers to form a central pore (shown as green arrow). **(D)** Side view of the structure of the human AQP1 monomer, which shows six transmembrane α-helices (TM1-6) including pseudo TM (LE and LB) that are connected with five different loops (A–E). **(E)** The top view of the human AQP1 is shown. The residues (F56, H180, C189, and Arg195) in the ar/R constriction and two NPA motifs (yellow and cyan) are shown in sticks. **(F)** The top view of the AQP homotetramers with filled amino acid residues is shown. The pore of each monomer and the central pore of the homotetramers are shown as white circles.

Although the amino acid sequences differ substantially, the structure of AQPs is highly conserved having a common tetrameric arrangement; each subunit behaves as a functional channel (Murata et al., 2000; Gomes et al., 2009; Schenk et al., 2010; Azad et al., 2016, 2018). However, a fifth pore is formed in the center of the tetramer. Each monomer is constituted of six transmembrane (TM) α-helices (H1–H6) with five connecting loops (loops LA–LE) and cytoplasmic N- and C-termini and form an individual pore that specifies the transport activity (Figure 2). There are two main constrictions in the channel. The first constriction is formed by two highly conserved Asn-Pro-Ala (NPA) motifs on loops B and E that is involved in proton exclusion (Sui et al., 2001; Tajkhorshid et al., 2002). Both NPA motifs protrude into the membrane from opposite side and form the seventh pseudo TM helix. The second constriction, called the aromatic/arginine (ar/R) selectivity filter, is formed by four residues from helix H2 and H5, and loop E (LE1 and LE2) (Fu et al., 2000; Heymann and Engel, 2000; Sui et al., 2001; Tajkhorshid et al., 2002; Kitchen et al., 2019). Substitutions at this ar/R selectivity filter are thought to determine the broad spectrum of substrate conductance (Beitz et al., 2006; Azad et al., 2012). While all AQPs share the same structural core architecture, there are some distinct structural variations in loops and the N- and C-termini suggesting their functional and/or regulatory roles (Gonen and Walz, 2006; Azad et al., 2021).

## Tissue-Specific Distribution and Physiological Relevance of Human Aquaporins

The complete tissue-specific distribution of AQPs throughout the human body and their physiological relevance are summarized (Figure 1 and Table 1). Several AQP isoforms (AQP1, 3, 4, 5, 7, 8, 9, and 11) are expressed in the brain and nervous system, however, AQP1 and AQP4 show relatively higher expression in the brain (Filippidis et al., 2016; Shchepareva and Zakharova, 2020). AQP1 is expressed in epithelial cells of the choroid plexus and plays a role in cerebrospinal fluid (CSF) formation (Brown et al., 2004). AQP1 is also present in primary sensory neurons and is proposed to be involved in pain perception (Borsani, 2010; Zhang and Verkman, 2010). AQP4 is found at the border between parenchyma and fluid-filled compartments and, more specifically, in astrocyte cell membranes, ependymal cells, and osmosensory areas of the hypothalamus (Jung et al., 1994; Xu et al., 2017). Eight (AQP0, 1, 3, 4, 5, 7, 9, and 11) of the 13 human AQPs are found in the eye (Figure 1; Tran et al., 2013; Schey et al., 2014). These AQPs perform various physiological roles such as maintenance of transparent cornea and lens, corneal wound healing, maintaining tear film osmolarity and retinal homeostasis (Schey et al., 2014). Nine AQPs (AQP1, 2, 3, 4, 5, 6, 8, 10, and 11) have been reported in human ear which are involved in neuronal signal transduction, regulating cell movement and lipid metabolism as well as immunological functions (Jung et al., 2017). Six AQPs are found in the skin, which are distributed to epidermis (AQP1, 3, 7, and 10), dermis (AQP1, 3, and 5), and hypodermis (AQP7) (Patel et al., 2017). Skin AQPs are associated with skin hydration, cell proliferation and differentiation, migration, immunity, and wound healing (Boury-Jamot et al., 2009; Sebastian et al., 2015; Bollag et al., 2020). Specifically, AQP3 is involved in epidermal cell proliferation and migration; AQP1 controls water movement between blood and dermis to maintain hydration; AQP5, found in both epical and basolateral membranes of the sweat gland is involved in water secretion; AQP7 plays important role to release glycerol from adipocytes for energy production and AQP9 facilitates the transport of glycerol, urea, and H_2_O_2_ for defense mechanism (Patel et al., 2017).

**TABLE 1:** Human AQPs major characteristics from chromosomal location to physiological relevance with the pathophysiological phenotypes due to their dysregulation, mutation, and dysfunction.

**AQPs**	**Chromosomal location**	**Exon number**	**Permeability**	**Tissue expression**	**Physiological function**	**Pathophysiological phenotype due to dysregulation, mutation, and dysfunction of AQPs**	**References**
AQP0	12q13	8	Water, CO_2_, ascorbic acid, cations	Eye	Corneal and lens transparency and homeostasis	Cataract	Kannan et al., 2001; Verkman et al., 2008b; Day et al., 2014; Li and Wang, 2017; Laloux et al., 2018
AQP1	7p14	7	Water, monovalent cations, H_2_O_2_, CO_2_, NO, NH_3_	Brain, eye, kidney, trachea, heart, lung, gastrointestinal tract, salivary gland, pancreas, liver, ovary, testis, muscle, erythrocytes, spleen	Osmotic water flux in tissues including eye, brain (choroid plexus), kidney and the vascular system	Diuresis, reduced tumor angiogenesis, reduced intraocular pressure, reduced CSF secretion, reduced nociception, astrocytoma, cholangiocarcinoma; breast, cervical, colorectal, lung, ovarian, laryngeal and nasopharyngeal cancers	Boassa and Yool, 2003; Day et al., 2014; Li and Wang, 2017; Laloux et al., 2018
AQP2	12q13	4	Water	Kidney, ear, ductus deferens	Maintains urine concentration and water homeostasis in renal system	Nephrogenic diabetes insipidus; congestive heart failure, liver cirrhosis and pre-eclampsia	Radin et al., 2012; Li and Wang, 2017; Laloux et al., 2018
AQP3	9p13	6	Water, urea, glycerol, ammonia, silicon, arsenite, H_2_O_2_	Kidney, brain, trachea, heart, ovary, eye, salivary gland, gastrointestinal tract, liver, Respiratory tract, brain,	Water and glycerol channel, facilitates skin hydration and also involved in cell migration during wound healing	Diuresis, dry skin, reduced growth of skin tumors, impaired skin wound healing, impaired regeneration of colonic epithelium and impaired leukocyte function; colorectal, cervical, liver, lung, esophageal cancers	Day et al., 2014; Li and Wang, 2017; Marlar et al., 2017; Laloux et al., 2018
				erythrocyte, fat, spleen			
AQP4	18q22	6	Water, CO_2_	Brain, eye, kidney, salivary gland, heart, gastrointestinal tract, muscle, trachea	Controls brain and kidney water homeostasis; cell migration, brain edema, metabolism and cell homeostasis	Reduced cytotoxic or increased vasogenic CNS edema, accelerated obstructive hydrocephalus, increased seizure threshold and duration, deafness and anosmia; meningioma, astrocytoma, breast, and lung cancer	Day et al., 2014; Papadopoulos and Saadoun, 2015; Li and Wang, 2017; Laloux et al., 2018
AQP5	12q13	5	Water, H_2_O_2_, CO_2_	Salivary gland, eye, trachea, lung, gastrointestinal tract, ovary, kidney	Controls water homeostasis	Reduced saliva secretion, reduced airway submucosal secretion, thin cornea and reduced tear volume; ovarian, breast, colorectal, cervical, leukemia, liver, lung, esophageal cancers	Day et al., 2014; Direito et al., 2016; Li and Wang, 2017; Laloux et al., 2018
AQP6	12q13	4	Water, urea, anion, ammonia, CO_2_, glycerol	Brain, kidney	Glomerular filtration, tubular endocytosis, and acid-base metabolism	Not yet reported	Yasui et al., 1999; Day et al., 2014; Li and Wang, 2017; Laloux et al., 2018; Shchepareva and Zakharova, 2020
AQP7	9p13	10	Water, urea, glycerol, ammonia, arsenite, antimonite and silicon	Testis, heart, kidney, ovary, fat	Energy homeostasis; spermatogenesis; facilitates glycerol efflux from adipocytes, prevent intracellular glycerol, and triglyceride accumulation	Obesity, insulin resistance, and hyperglyceroluria	Verkman, 2012; Day et al., 2014; Li and Wang, 2017; Iena and Lebeck, 2018; Laloux et al., 2018
AQP8	16p12	6	Water, urea, ammonia, H_2_O_2_, and glycerol	Testis, liver, pancreas, ovary, lung, and kidney	Water trafficking from lumen to the interstitium by a transcellular route; modulates membrane water permeability	Astrocytoma, colorectal and liver cancers, cerebral edema, inflammatory bowel diseases	Day et al., 2014; Pelagalli et al., 2016; Laloux et al., 2018; Prata et al., 2019; Escudero-Hernández et al., 2020; Shchepareva and Zakharova, 2020
AQP9	15q22	6	Water, urea, glycerol, arsenite, H_2_O_2_, antimonite, silicon, lactic acid, and CO_2_	Liver, spleen, testis, ovary,	Regulates neutrophil cell migration; maintains energy balance in neurons by enabling the diffusion of glycerol and monocarboxylates; metabolic regulation in diabetes and obesity	Hyperglycerolaemia and reduced red cell glycerol permeability; astrocytoma, liver, and ovarian cancer	Li and Wang, 2017; Laloux et al., 2018; Shchepareva and Zakharova, 2020
				leukocyte			
AQP10	1q21	6	Water, urea, glycerol, arsenic, antimonite, silicon	Intestine, keratinocytes of the epidermis, adipose tissue	Carrier and channel for glycerol and other solutes transport	Not yet reported	Day et al., 2014; da Silva and Soveral, 2017; Li and Wang, 2017; Laloux et al., 2018
AQP11	11q13	3	Water and glycerol	Testis, heart, kidney, ovary, muscle, gastrointestinal tract, leukocytes, liver, and brain	Maintains normal urine concentration and renal function	Polycystic kidneys and	Day et al., 2014; Li and Wang, 2017; Laloux et al., 2018; Han et al., 2019
						hepatocyte vacuolization,	
						chronic kidney disease	
AQP12	2q37	4	Not yet reported	Pancreas	Not yet reported	Pancreatitis	Ohta et al., 2009; Day et al., 2014; Li and Wang, 2017; Laloux et al., 2018

Nine AQPs (AQP1, 2, 3, 4, 5, 6, 7, 8, and 11) are expressed in kidneys to maintain the body water homeostasis, tissue development together with metamorphosis of various substances. Kidney AQPs play important roles in both the short- and long-term regulation of water balance (He and Yang, 2019; Su et al., 2020). AQP1 is one of the most abundant AQPs in the kidney, which is involved in water reabsorption in various segment of the kidney such as loop of Henle, apical and basolateral membranes of proximal tubules and vasa recta (Su et al., 2020). The remaining kidney AQPs are found in collecting duct and proximal tubule (Matsuzaki et al., 2017). Eight AQPs namely AQP1, 3, 4, 5, 7, 9, 10, and 11 are found in cardiovascular system (Figure 1). These AQP homologs located to the heart, endothelial cells, and vascular smooth muscle participate in the transportation of water, glycerol, and lactic acid, which play pivotal roles in cardiac physiology (Butler et al., 2006; Verkerk et al., 2019; Montiel et al., 2020). Ten AQPs (AQP0, 1, 2, 3, 4, 5, 7, 8, 9, and 11) expressed in male reproductive system (Figure 1) act as water, glycerol and ion channel during various process involved such as spermatogenesis, sperm osmoadaptation, and folliculogenesis (Day et al., 2014; Carrageta et al., 2020). These AQPs are localized in various sections of the male reproductive system including epididymis, testis, efferent ducts, prostate, seminiferous tubules, seminal vesicles, germ cells, sertoli cells, spermatids, spermatozoa, and vas deferens (Carrageta et al., 2020). AQP1, 2, 3, 4, 5, 6, 7, 8, 9, 11, and 12 have been shown to be expressed in different segments of the female reproductive system such as vagina, cervix, uterus, oviduct, ovary, oocyte, embryo, amnion, and chorion (Kordowitzki et al., 2020). Various types of biological process of the female reproductive system are conducted through AQPs like vaginal lubrication, sperm movement and implantation, and follicular development (Kordowitzki et al., 2020). In human digestive system, eleven AQP isoforms are found except AQP6 and 12 (Zhu et al., 2016; Liao et al., 2020). The most prolific AQP in the digestive system is AQP1 which is found in all organs including small and large intestine, salivary gland, oral cavity, esophagus, stomach, liver, gall bladder, bile duct, and pancreas (Zhu et al., 2016; Liao et al., 2020). Their extensive distribution indicates their wide range of biological functions like water and glycerol transportation, saliva excretion, adipose absorption, pancreatic excretion, cellular proliferation, migration, invasion, apoptosis, gastric acid secretion, and cellular signaling (Liao et al., 2020). AQP1, 3, and 4 are expressed in skeletal muscle (Figure 1). The localization of AQP1 and AQP4 within the muscle tissue suggests their possible function as water channel during muscular contraction (Au et al., 2004; Frigeri et al., 2004; Mobasheri et al., 2004). AQP1 and AQP3 identified within the nucleus pulposus cells of the human intervertebral disk might be involved in cell swelling during mechanistic load (Frigeri et al., 2004; Rutkovskiy et al., 2013). AQP9 is found in osteoclast cells, astrocytes, and catecholaminergic neurons; however, it is not essential for osteoclast function or differentiation under normal physiological conditions (Rutkovskiy et al., 2013; Halsey et al., 2018).

## Functional Diversity and Regulation of Human Aquaporins

Aquaporins facilitate the transport of water and other small solutes such as glycerol, nitrate, urea, ammonia, H_2_O_2_, CO_2_, O_2_, arsenic, antimony, silicon, sodium ion, and aluminum malate (Table 1, Liu et al., 2002; Carbrey and Agre, 2009; Garneau et al., 2015; Byrt et al., 2017; Wang et al., 2017); some are physiologically important and some are toxic heavy metals (Bienert and Chaumont, 2011; Mukhopadhyay et al., 2014; Perez Di Giorgio et al., 2014; Finn and Cerdà, 2015). While AQPs play important physiological roles in humans, their mutations, malfunction or dysfunction, and dysregulation generate a lot of pathophysiological phenotypes leading to many severe diseases (Table 1). The transcellular water movement is strongly organized by the amount and activity of AQPs present in cellular membranes, and the movement of water molecules is occurred based on the osmotic gradient (Chaumont and Tyerman, 2014). All thirteen AQPs reported in human (AQP0–12) to date, are usually classified into three groups: orthodox AQPs (AQP0, 1, 2, 4, 5, 6, and 8), aquaglyceroporins (AQP3, 7, 9, and 10), and the unorthodox AQPs also called superaquaporins (AQP11 and 12) (Soto et al., 2012; Abascal et al., 2014; Finn et al., 2014; Finn and Cerdà, 2015). It has been reported that AQP3, 8, 9, and 11 are capable of facilitating the diffusion of H_2_O_2_ (Miller et al., 2010; Hara-Chikuma et al., 2012a; Bienert and Chaumont, 2014; Medraño-Fernandez et al., 2016; Watanabe et al., 2016; Bestetti et al., 2020; Hara-Chikuma et al., 2020), and the AQP-facilitated H_2_O_2_ transport is involved in psoriasis development and teleost spermatozoon motility (Chauvigné et al., 2015; Hara-Chikuma et al., 2015; Thiagarajah et al., 2017). AQP1, 3, 6, 7, 8, 9, and 10 are permeable to ammonia (Litman et al., 2009; Finn and Cerdà, 2015). The ammonia transporting mechanism is not yet fully resolved but some experimental data show that ammonia is transported in its neutral form, NH_3_. Aquaglyceroporins (AQP3, 7, 9, and 10) and AQP6 are also reported to transport the urea, which might be involved in energy metabolism (Litman et al., 2009). Human AQPs have been shown to facilitate diffusion of arsenite at neutral pH. Arsenite is used as a chemotherapeutic agent for acute promyelocytic leukemia and diseases caused by protozoan parasites because of its toxic properties to the cells (Rosen and Tamás, 2010). AQP3, 7, 9, and 10 have also been reported to facilitate the transport of antimonite (Liu et al., 2002). The overexpression of AQP has been shown to increase the uptake of antimonite and hypersensitivity of leukemia as well as lung adenocarcinoma cell lines (Bhattacharjee et al., 2004; Mukhopadhyay et al., 2014). Several studies reported that AQP0, 1, 4, 5, and 6 are involved in CO_2_ transport (Table 1). Recently, AQP1 has been shown to facilitate O_2_ transport by the spectrophotometric assay using yeast spheroplasts (Zwiazek et al., 2017). AQP1 is also reported to transport the nitric oxide (NO) (Herrera et al., 2006). In *Xenopus* oocytes, human AQP1 led to a PKA-activated and/or cGMP-activated ion permeability by its phosphorylation (Yool et al., 1996). Additionally, AQP6 acts as an anion channel, which is activated by low pH or Hg^2+^ (Hazama et al., 2002). The ion channel activity of AQPs could play a vital role for several pathophysiological processes including tumor progression (Saadoun et al., 2005a). It is speculated that the central pore of AQP tetramer could be the channel for transporting gases and ions (Hub and de Groot, 2006; Hub and de Groot, 2008). However, gases or ions permeation activities of AQPs are still controversial and required to be further investigated to figure out the physiological relevance of these new putative substrates. Two orthodox AQPs (AQP1 and 5) and four aquaglyceroporins (AQP3, 7, 9, and 10) expressed in the skin facilitate the transport of water and some other small solutes such as glycerol, which play critical roles in regulating numerous skin parameters (Boury-Jamot et al., 2006, 2009; Patel et al., 2017).

Aquaporins are synthesized and inserted in the endoplasmic reticulum membrane and finally localized to the target membrane via the secretory Sec61 translocon present in all domains of life (Pitonzo and Skach, 2006; Azad et al., 2011a). Their activity needs to be properly regulated in the target membrane to keep the nutrient homeostasis in cells (Chaumont and Tyerman, 2014). Presence of several AQPs in human suggests their multiple functions in cellular and organs levels. Some AQPs are present in intracellular vesicles, but can relocate later in the PM. For instance, AQP2 and AQP8 relocate to the PM from intracellular vesicles in renal collecting ducts and rat hepatocytes in response to vasopressin and cAMP, respectively (Marples et al., 1995; Nielsen et al., 1995). Some AQPs have intracellular functions, i.e., AQP8 and 9 were detected in the mitochondrial membrane (Amiry-Moghaddam et al., 2005; Calamita et al., 2005; Molinas et al., 2012). AQP trafficking is very dynamic process that at first targets the PM and removes it from the membrane for degradation or recycling in the endosome. Interestingly, the mislocalization of AQPs could lead to some human congenital disorders i.e., nephrogenic diabetes insipidus (NDI) is caused by AQP2 mislocalization (Bichet et al., 2012). The localization of AQP4, the predominant AQP isoform in the brain, relies on two C-terminus motifs namely a tyrosine motif (YxxΦ; Φ, V/L/I/F) and a dileucine-like motif. Upon mutation of any of the two motifs to alanine, AQP4 was relocated to the apical membrane instead of the basolateral membrane (Matter et al., 1992; Hunziker and Fumey, 1994). Phosphorylation is one of the important regulatory mechanisms that is involved in both gating and trafficking of AQPs (Li and Wang, 2017; Santoni, 2017; Takano et al., 2017). The precise relocalization of AQP2 to the PM from intracellular vesicles under vasopressin treatment involves the phosphorylation of the C-terminus Ser256 (Fushimi et al., 1997; van Balkom et al., 2002). However, the phosphorylation of Ser261 was found in vesicle-localized AQP2, which need to be dephosphorylated for PM relocation (Hoffert et al., 2007; Lu et al., 2008; Tamma et al., 2011). On the other hand, AQPs recycling is an important process for cells, and AQPs have been shown to be ubiquitinated to control its degradation (Leitch et al., 2001). For example, AQP2 is ubiquitinated at Lys270, which triggers its internalization in kidney collecting duct cells (Kamsteeg et al., 2006).

## Roles of Aquaporins in Infectious Diseases

Pathogenic bacteria, fungi, virus, and parasites can cause systemic infection and spread to different organs of the body (Figure 1). Sepsis, the most common cause of death, appears as a clinical syndrome following a local infection accompanied with an appropriate inflammatory response and becomes amplified with detrimental effects on the whole body leading to dysfunction of numerous organs (Keel, 2014). Sepsis displayed by two or more systemic inflammatory response syndrome (SIRS) increases the risk of secondary infection. However, the immune system becomes stimulated in response to both local and systemic infection, which may influence the expression and function of AQPs to maintain cellular and tissue homeostasis (Table 2).

**TABLE 2:** Regulation of expression of AQPs during infectious diseases.

**AQPs**	**Tissue**	**Infectious agents**	**Regulation of expression**	**Disease**	**Experimental species**	**References**
AQP1	Leukocytes	Lipopolysaccharide (LPS)	**↑**	Sepsis	Human	Vassiliou et al., 2013
	THP-1 cells	LPS, Terbutaline	**↑**	Endotoxemia	Mice	Rump et al., 2013
	Kidney	LPS	Deficient	Acute kidney injury (AKI)	Mice	Montiel et al., 2014
	Lung epithelia cells	Adenovirus	**↓**	Bronchitis	Mouse	Towne et al., 2000
	Human liver tissues	Hepatitis B virus	**↑**	Liver cirrhosis	Human	Xian et al., 2009
	Rotavirus		**↓**	Infantile gastroenteritis	Mouse	Cao M. et al., 2014
	Heart	LPS	Deficient	Cardiac hypertrophy	Mice	Madonna et al., 2012
	Cervix	HPV	**↑**	Cervical cancer	Human	Chen et al., 2014
	Lung cells	LPS	**↓**	Sepsis	Rat	Tao et al., 2016
AQP2	Kidney	LPS	**↓**	Endotoxemia-induced AKI	Rat	Grinevich et al., 2004; Chagnon et al., 2008; Olesen et al., 2009
	Colon	*E. coli*	**Mislocalization**	Diarrhea	Mouse	Zhu et al., 2016
	Colon	*C. rodentium*	**Mislocalization**	Diarrhea	Mice	Guttman et al., 2007
	Kidney	LPS	**↓**	AKI	Rat	Chagnon et al., 2008; Cui et al., 2011; Rodrigues et al., 2012; Suh et al., 2015
AQP3	THP-1 cells	LPS	**↓**	Diarrhea	Human colon epithelial cells	Li et al., 2015
	Duodenum	*V. cholera*	**↓**	Diarrhea	Human epithelial cells	Flach et al., 2007
	Squamous epithelial cells, Lymph node	Epstein-Barr virus		Nasopharyngeal carcinoma and lymphoma	Human	Wang J. et al., 2018
	Colon	AQP inhibitor	**↓**	Diarrhea	Rat	Ikarashi et al., 2012
	HT-29 cells	LPS	**Inhibition**	Diarrhea	Rat	Li et al., 2015
	Colon	*E. coli*	**Mislocalization**	Diarrhea	Mouse	Zhu et al., 2016
	Cervix	HPV	**↑**	Cervical cancer	Human	Chen et al., 2014
	Gastric mucosa	*H. pylori*	**↑**	Gastric cancer	Rat	Wang G. et al., 2012; Wen et al., 2018
	Colon	HgCl_2_ and CuSO_4_	**Inhibition**	Diarrhea	Rat	Ikarashi et al., 2012
	Colon	*C. rodentium*	**Mislocalization**	Diarrhea	Mice	Guttman et al., 2007
	Liver	*Plasmodium* spp.	**Relocalization**	Malaria	Human hepatoma cells, Rat	Bietz et al., 2009; Posfai et al., 2018, 2020
AQP4	Brain	LPS	**↑**	Brain edema	Mice	Du et al., 2014
	Brain	Endotoxin, Systemic sepsis	↑	Brain edema	Animals	Davies, 2002
	Intestinal epithelial cells	Rotavirus	**↓**	Infantile gastroenteritis	Mouse	Cao M. et al., 2014
	Brain	Dengue virus	**↑**Anti-AQP4	NMOSD	Human	Lana-Peixoto et al., 2018
	Astrocyte	HIV	**↑**	HIV-Dementia	Human	St Hillaire et al., 2005
	Brain	Herpes simplex virus	**↓**	Encephalitis	Mice	Martinez Torres et al., 2007
	Astrocyte	SHIV	**↓**	Cortical degeneration	Macaques	Xing et al., 2017
	Brain (Protective role)	*P. berghei*	**↓**	Cerebral malaria	Mice	Promeneur et al., 2013
	Liver	*S. japonicum*	**↓**	Schistosomiasis	Mice	Zhang et al., 2015
AQP5	THP-1 cells	LPS	**↓**	Sepsis	Mice	Rump et al., 2013
	Bronchial epithelial cells	LPS	**↓**	Bronchitis	Human bronchial epithelial cells	Shen et al., 2012
	Lung epithelial cells	Adenovirus	**↓**	edema	Mouse	Towne et al., 2000
	Lung cells	LPS	**↓**	Sepsis	Rat	Tao et al., 2016
AQP6	C3H10T1/2 chimeric cells	Hazara virus	**↓**	Severe hemorrhagic manifestations	Human	Molinas et al., 2016
AQP7	Colons	Dextran sodium sulfate	**↓**	Colonic injury	Mouse, Human	Hardin et al., 2004
AQP8	Lung cells	LPS	**ua**	Pulmonary injury	Rat	Kang et al., 2017
	Jejunum	*E. coli* and LPS	**↓**	Diarrhea	Piglet	Loos et al., 2012; Hou et al., 2013
	Intestinal epithelial cells	Rotavirus	**↓**	Infantile gastroenteritis	Mouse	Cao M. et al., 2014
	Small intestine	cholera toxin	**↓**	Diarrhea	Rat	Flach et al., 2004
			**↓**	Colonic injury	Mouse, Human	Hardin et al., 2004
	Sperm cells	Human papilloma virus	**Inhibition**	Male sub-fertility	Human	Pellavio et al., 2020
	Liver	LPS	**↓**	Cholestasis	Rat	Lehmann et al., 2008
AQP9	Lung cells	LPS	**ua**	Pulmonary injury	Rat	Kang et al., 2017
	Brain	LPS	**↑**	Edema	Rat	Wang et al., 2009
	Leukocyte	LPS	**↑**	Endotoximia	Human	Talwar et al., 2006
	Leukocyte	*N*-formylmethiony lleucylphenylalanine	**↑**	Systemic inflammatory response syndrome (SIRS)	Human	Matsushima et al., 2014
	Brain	HSV-1	**↑**	Encephalitis	Rat	Jennische et al., 2015; Bello-Morales et al., 2020
	Macrophages	*P. aeruginosa*	**↑**	Bacterial infection	Human	Holm et al., 2015, 2016
	Blood	Bacterial LPS	**↑**	Endocarditis	Human	Talwar et al., 2006; Thuny et al., 2012
	Liver	*Plasmodium* spp.	**Relocalization**	Malaria	Mice	Liu et al., 2007
AQP10	Duodenum	Cholera toxin	**↓**	Diarrhea	Human	Flach et al., 2007

*↑, up-regulation; ↓, down-regulation; ua, unaffected.*

### Bacterial Infections and AQPs

Bacteremia or septicemia occurs when bacteria spread in the bloodstream following a bacterial infection elsewhere in the body, such as the skin or lungs and so on. This is very precarious as the bacteria including their toxins can be carried through the bloodstream to the entire body. Bacteria are a prominent source of both endotoxins and exotoxins, which lead to severe sepsis and death (Opal, 2007; Ramachandran, 2014).

Bacterial endotoxins such as lipopolysaccharide (LPS) serve as an important stimulator to regulate the expression of human AQPs (Rump and Adamzik, 2018). Vassiliou et al. (2013) reported that expression of AQP1 is increased twofold in leukocytes in response to sepsis in patients of intensive care unit (ICU) (Table 2). They showed *in vitro* that LPS stimulates polymorphonuclear granulocytes (PMNs) for increased expression of AQP1, and the LPS-stimulated PMNs transiently increased cell volume upon hypotonic treatment. LPS exposure to cell lines also increased the expression of AQP1 but decreased that of AQP3 and AQP5 (Rump et al., 2013; Li et al., 2015). However, another study showed that LPS decreased the expression of AQP5, but not AQP3 and AQP4 in human primary bronchial epithelial cells (Shen et al., 2012). In LPS induced-endotoxemia, expression of AQP1 in the kidney and heart might have a protective role because mice deficient in AQP1 revealed predisposition to endotoxemia-induced acute kidney injury (AKI) (Wang et al., 2008) and cardiac hypertrophy (Madonna et al., 2012; Montiel et al., 2014), respectively (Table 2). However, upon exposure of LPS, expression of AQP1 and AQP5 is decreased in lung cells but that of AQP8 and AQP9 remains unaffected (Fisher et al., 2012; Ma and Liu, 2013; Tao et al., 2016; Kang et al., 2017). In animal models, several studies showed that LPS-induced endotoxemia down-regulated AQP2 expression in kidney (Grinevich et al., 2004; Chagnon et al., 2008; Olesen et al., 2009). However, pretreatment of model animals with continuous erythropoietin receptor activator or propofol or α-lipoic acid prevents AQP2 down-regulation and protects against endotoxemia-induced AKI (Chagnon et al., 2008; Cui et al., 2011; Rodrigues et al., 2012; Suh et al., 2015). While LPS-induced TNF-α down-regulates AQP8 in rat liver (Lehmann et al., 2008), systemic bacterial LPS stimulates up-regulation of AQP9 in rat brain (Wang et al., 2009). The LPS-induced TNF-α-mediated down-regulation of AQP8 in hepatocytes has been proposed as a potential molecular mechanism for pathogenesis of sepsis-associated cholestasis (Lehmann et al., 2008; Marinelli et al., 2011). The up-regulation of AQP9 expression is suggested to exert important roles in water transport associated with the pathophysiology of brain edema induced by LPS injection (Wang et al., 2009). Furthermore, the up-regulation of AQP4 in brain in response to LPS may aggravate the brain edema (Du et al., 2014). AQPs are supposed to play important role in septic encephalopathy associated with brain edema (Davies, 2002). It has been shown that AQP9 is up-regulated in blood leukocytes in response to intravenous bacterial LPS (Talwar et al., 2006). The expression of this homolog is increased in patients SIRS compared to healthy individuals (Matsushima et al., 2014), and in infective endocarditis patients (Thuny et al., 2012). Furthermore, *Pseudomonas aeruginosa* produces quorum sensing molecules and induce increased expression, distribution and reorganization of AQP9 in macrophages by changing the cell volume accompanied water fluxes across cell membrane through AQP9 (Holm et al., 2015, 2016) (Table 2). These events affect cell migration and phagocytosis which may have influence on the immunity, outcome of infection, inflammation, and thus disease development (Holm et al., 2015, 2016).

Expression of several AQPs is changed during diarrhea caused by enteropathogenic and enterohemorrhagic *Escherichia coli*, their LPS and enterotoxin of *Vibrio cholerae* (Flach et al., 2007; Zhu et al., 2016). Bacteria introduced into the body through oral ingestion of contaminated water or food colonize in the intestinal epithelial cells and employ different effector proteins in the host and alter the normal function of cells and cause diarrhea. Along with several other onset mechanisms of diarrhea such as reduction of absorptive surface area due to the effacement of microvilli (Donnenberg et al., 1993), altered ion channel activity (Guttman et al., 2006) or tight junction disruption resulting in loss of barrier function (Dean et al., 2006), AQPs are thought to contribute to the development of diarrhea after pathogen invasion (Guttman et al., 2007). At least six AQPs (AQP1, 3, 4, 5, 8, and 9) are localized in the apical and basolateral membranes of the human colon (Liao et al., 2020), which might be involved in the extraction of water and electrolyte from solid waste back into the body and thus dehydrate feces (Ma and Verkman, 1999). Inhibition, reduced expression and mislocalization of AQPs in the human colon might have roles in onset of diarrhea. Down-regulation of AQP3 in the duodenum is associated with acute diarrhea caused by *V. cholera* (Flach et al., 2007). In rat, the inhibition of AQP3 by HgCl_2_ and CuSO_4_ induced colon diarrhea keeping its expression static both in mRNA and protein levels (Ikarashi et al., 2012) (Table 2). In the same model, cholera toxin reduced the expression of mucosal AQP8 in the small intestine to cause diarrhea (Flach et al., 2004). Alteration in AQPs localization might be important in the onset of diarrhea. A mouse model infected with *Citrobacter rodentium* demonstrated that the AQP2 and AQP3 mislocalized within cytoplasm of colonocytes rather than their normal location in the cell membranes, and the infected mice eventually showed phenotypic diarrhea (Guttman et al., 2007). In piglet model, enterotoxigenic *E. coli* and LPS reduced expression of mucosal AQP8 in the jejunum and developed diarrhea (Loos et al., 2012; Hou et al., 2013). Several studies showed that some substances such as emodin, berberine, and MgSO_4_ may change and regulate water transport and absorption activity of AQPs involving the cAMP-dependent/p-CREB signaling pathway (Ikarashi et al., 2011; Zhang et al., 2012b; Liu et al., 2014; Zheng et al., 2014).

*Helicobacter pylori* regarded as the class I carcinogen in the human stomach and are responsible for the development of gastric carcinomas in the distal portion of the stomach (Konturek et al., 2009; Shin et al., 2010). Gastric carcinogenesis might be initiated from combination of events including increased DNA damage, decreased DNA repair activity and genetic instability in gastric cells due to mutation in mitochondrial DNA following *H. pylori* infection (Machado et al., 2010). *H. pylori* strains produce various toxins including cytotoxin-associated gene A (CagA) and vacuolating cytotoxin (VacA) to promote carcinogenesis (Jang et al., 2010). AQPs have shown its potential roles in tumor cell migration and proliferation (Hu and Verkman, 2006). A study showed that AQP3 and 5 expressed in higher levels in gastric carcinomas than the normal mucosa were associated with lymph node metastasis and lymphovascular invasion (Shen et al., 2010). Another independent study revealed that higher expression of AQP3 correlated with *H. pylori* infection status in gastric cancer tissues in comparison with the normal mucosa (Wang G. et al., 2012). This study further showed that *H. pylori* infection increased expression of AQP3 in gastric mucosa in a Sprague Dawley rat model. Recently, Wen et al. (2018) proposed that the up-regulation of AQP3 in gastric carcinoma caused by *H. pylori* was associated with the activation of reactive oxygen species (ROS) pathway (Table 2).

Although there are no enough studies associating infectious colitis with AQPs, a study showed a significant reduction in the expression of AQP7 and 8 both in protein and mRNA levels (Hardin et al., 2004). The down regulation of AQPs in infectious colitis may be associated with the microbiome of the host (Wang et al., 2019). Mice deficient in AQP4 alleviates experimental colitis, and it has been shown that there is significant difference in the microbiome of AQP4-knockout mice compared to the WT mice (Wang et al., 2019). The perturbation in the microbiome is further connected with the levels of inflammatory molecules such as IL-6, IL-10, and TNF. AQPs may modulate intestinal inflammation through the regulation of the abundance of intestinal microbiota (Wang et al., 2019).

### Viral Infection and AQPs

As mentioned earlier, AQPs play important roles in cell volume, cell migration, organelle physiology through regulating cellular and tissue water homeostasis (Verkman, 2005; Verkman et al., 2008a; Karlsson et al., 2013a,b; Holm et al., 2015). Several studies have been done showing the involvement of AQPs with the viral infections (Table 2).

Very recently, it has been shown that human papilloma virus (HPV) infection affects the expression and functionality of AQP8, and lead to male sub-fertility (Pellavio et al., 2020). AQP8 expressed both in the PM and intragranular membranes of sperm cells has been suggested to facilitate the transport of water and H_2_O_2_, and the AQP8-mediated H_2_O_2_ permeability might be involved in ROS scavenging and thus detoxification (Laforenza et al., 2016; Pellavio et al., 2020). However, the direct interaction between the capsid protein L1 of the HPV and AQP8 alters the water and H_2_O_2_ permeability (Pellavio et al., 2020). The HPV is a common causative agent of cancer (Tornesello and Buonaguro, 2020). The HPV infection status and the overexpression of AQP1 and 3 are associated with poor outcome of cervical cancer (Table 2); however, their overexpression levels are not independent risk factors which are related with the prognosis of cervical carcinoma (Chen et al., 2014). These AQPs might have influenced the prognosis of cervical cancer by promoting HPV invasion, tumor growth and lymphatic metastasis (Chen et al., 2014).

In experimental mice model, Herpes simplex virus (HSV) decreased AQP4 in the acute phase of infection but increased its expression along with AQP1 in the long-term infection (Martinez Torres et al., 2007) (Table 2). As the AQP4 is widely expressed in the brain-blood interfaces (Manley et al., 2000), it might have regulated the pathophysiology of the acute and chronic HSV encephalitis (HSVE), and the modulation of AQP4 could be a potential target for treatment of HSVE (Martinez Torres et al., 2007). AQP4 antibody and concomitant HSV-1 and HSV-1 infections have been associated with myeloradiculitis and encephalopathy; consequently AQP4 antibody test could be routinely used for HSV infection-mediated encephalopathy or neuromyelitis optica and autoimmune AQP4 channelopathy (Marin Collazo et al., 2018; Peng et al., 2018). Immunohistochemistry revealed that the neuronal cell population that conveyed HSV-1 infection through the anterior commissure in a rat model was positive of AQP9 (Jennische et al., 2015). This study reports that the CSF samples from HSVE patients showed higher levels of AQP9 compared to controls, suggesting AQP9 to be involved in viral spreading and pathogenesis of HSVE (Jennische et al., 2015; Bello-Morales et al., 2020).

Crimean–Congo hemorrhagic fever virus (CCHFV), a highly pathogenic arthropod-borne agent, causes infectious disease with multiple organ failure and severe hemorrhage in vascular system in humans. Vascular permeability plays a pivotal role in the development of this disease which is regulated by AQPs. A study showed that infection by Hazara virus, a model for CCHFV, reduced cellular and prenuclear AQP6 distribution and changed the cell volume (Molinas et al., 2016) (Table 2). The viral infection down-regulated the expression of AQP6 at mRNA levels in human cells. However, overexpression of AQP8 in human cells displayed protective roles by decreasing the viral infectivity (Molinas et al., 2016). Recently, it has been reported that Epstein-Barr virus (EBV)-associated nasopharyngeal carcinoma (EBVaNPC) and lymphoma (EBVaL) are concomitant with the single-nucleotide polymorphism locus of AQP3 (rs2231231) (Wang J. et al., 2018). This study reveals that the homozygous genotype is commonly detected in patients with EBVaNPC and EBVaL. Neurological complications with the phenotype of neuromyelitis optica spectrum disorder (NMOSD) have been described in two patients with dengue fever following infection with the Dengue virus (Lana-Peixoto et al., 2018). Both patients showing brainstem symptoms or isolated unilateral optic neuritis were positive for serum AQP4 antibody (Lana-Peixoto et al., 2018), and AQP4 antibodies have been proposed as a pathogenic and diagnostic biomarker for NMOSD (Berger et al., 2017). A very recent case report concludes that AQP4 antibody positive NMOSD co-exists with varicella-zoster virus radiculomyelitis (Eguchi et al., 2020). It is reported that decrease of AQP4 is concomitant with astrocyte dysfunction for pathogenesis of cortical degeneration in HIV-associated neurocognitive disorders (Xing et al., 2017); however, increase of this AQP homolog is associated with HIV dementia (St Hillaire et al., 2005).

A study in mouse model infected with Rotavirus demonstrates that the down-regulation of AQP1, 4, and 8 are associated with Rotavirus diarrhea, a major worldwide cause of infantile gastroenteritis (Cao M. et al., 2014). Likewise, pulmonary adenoviral infections in mice down-regulated AQP1 and AQP5 in lung epithelia cells (Towne et al., 2000). However, higher expression of AQP1 at both mRNA and protein levels was reported in cirrhotic liver tissues in patients infected with Hepatitis B virus compared to normal tissues (Xian et al., 2009). Interestingly, *Chlorella* virus MT325 has been reported to have an AQP gene, *aqpv1* which functions as an aquaglyceroporin in *Xenopus* oocytes (Gazzarrini et al., 2006). Co-expression of this viral AQP with its potassium channel in the *Xenopus* oocytes synergistically increases the water transport that could have pathophysiological relevance. However, no further investigation has been done with this viral AQP to confirm the pathophysiological relevance and other viral AQP is not yet reported.

### Parasitic Infection and AQPs

Organisms known as parasites depend on a host for feeding and reproduction include members of numerous taxa mainly protozoa, helminths, and arthropods. In a global context, the most important human protozoan parasites including *Leishmania* and *Plasmodium* are transmitted by bloodsucking arthropods, and *Toxoplasma* is soil or food-born. Parasites harm their hosts by causing serious disease or even death (Ni et al., 2017; Walochnik et al., 2017).

*Plasmodium* parasite causing malaria disease in human infects the erythrocyte by passing through the multiple hepatocyte cells in the liver. In the liver stage, the parasite is matured to its infectious form merozoite and released to the blood stream to cause the symptoms of malaria. The obligatory liver stage is the critical part of this parasite invasion and AQP plays positive role in the nutrient transfer to the parasite from the host. During invasion in hepatocyte and blood cells, a parasitophorous vacuole membrane (PVM) derived from the host is formed around the parasite and act as an interface between the host cell and the parasite (Sherling and Ooij, 2016; Nyboer et al., 2018). The PVM is a prerequisite for *Plasmodium* growth and development. A study showed AQP3 localized in the PVM, facilitated the transport of water and glycerol to *Plasmodium*, and identified it as essential to parasite development in hepatoma cells (Posfai et al., 2018). Several studies revealed that AQP3 is recruited to the PVM in liver-stage *P. berghei* schizonts and blood-stage *P. falciparum* and *P. vivax* schizonts, and is thought to facilitate the transport of water or nutrients between the parasites and the host cell (Bietz et al., 2009; Posfai et al., 2018, 2020). These studies discussed that a significantly induced AQP3 expression was observed in the infected hepatocytes compared to the uninfected ones (Table 2). However, deletion of AQP3 or treatment with AQP3 inhibitor can reduce the pathogen burden in the liver and blood stage of infection (Bietz et al., 2009; Posfai et al., 2018, 2020). Therefore, AQP3 might have played important role in *Plasmodium* infection and its modulation would facilitate disease control efforts. Although human and rats have AQP3, the mice lack this AQP homolog but possess AQP9 that plays role as an aquaglyceroporin. AQP9-deficient mice missed the function of glycerol channel in their erythrocytes, and infected with *P. berghei* survived longer compared to the WT mice (Liu et al., 2007). This study suggests that the transport pathway through AQP9 may contribute to the virulence of intraerythrocytic stages of malarial infection (Table 2). However, AQP4 has been suggested to play protective roles in murine cerebral malaria (Promeneur et al., 2007). This study showed that mice infected with *P. berghei* and showing cerebral malaria displayed a reduction of brain AQP4 at transcript and protein levels. In comparison with the WT mice, AQP4-knockout mice showed earlier appearance and more severe signs of cerebral malaria with greater brain edema. Another independent study revealed that AQP4 has association with the immunoregulation of the liver granuloma formation during schistosomiasis developed by a parasite *Schistosoma japonicum* in mice (Zhang et al., 2015) (Table 2). *S. japonicum*-infected AQP4-null mice with schistosomiasis exhibited greater granulomatous response with increased accumulation of eosinophils, macrophages and Th2 but reduced Th1 and T regulatory cells generation. Upregulation of Th2 and Th17 cells and downregulation of Th1 and T regulatory cells are the hallmarks of granuloma formation in schistosomiasis (Baumgart et al., 2006; Turner et al., 2011; Wen et al., 2011).

## Involvement of Aquaporins in Non-Infectious Diseases

The AQPs are involved in a wide range of human non-infectious diseases including cancer, cerebral edema and ischemic stroke, renal dysfunction, glaucoma, epilepsy, and obesity (Table 1). Defects in *AQP* genes may lead to several human diseases like hereditary NDI (Deen et al., 1994) and congenital cataracts (Verkman and Mitra, 2000).

### AQPs in Cancer

Aquaporins play a key role in cancer pathogenesis and several tumor-related processes including tumor edema, tumor cell migration/invasion, tumor proliferation and angiogenesis (Saadoun et al., 2002a,b, 2005a,b; Hara-Chikuma and Verkman, 2006; Auguste et al., 2007; Warth et al., 2007). About twenty types of tumors have been shown to be associated with the expression of AQPs (Khajah and Luqmani, 2016). Astrocytomas are infiltrating brain tumors that arise from astrocytes, which are histologically classified as grades II, III, or IV, with the most malignant grade IV also termed as the glioblastoma. In human astrocytomas, the expression levels of AQP1, 4, and 9 are largely increased (Saadoun et al., 2002a,b; Nico et al., 2009; El Hindy et al., 2013; Jelen et al., 2013), while the level of AQP1 was shown to be either increased (Mazal et al., 2005) or decreased (Aishima et al., 2007) in cholangiocarcinoma. In comparison with the normal tissues, AQP1, 3, and 5 are up-regulated in colorectal (Moon et al., 2003; Yoshida et al., 2013; Shi et al., 2014), cervical (Zhang et al., 2012a), and breast cancers (Kang et al., 2015; Qin et al., 2016) but AQP8 is down-regulated in the former case (Wang W. et al., 2012). Higher levels of AQP3 and 5, and lower levels of AQP8 and 9 are reported in liver cancer (Jablonski et al., 2007; Guo et al., 2013). Several studies show that the overexpression of AQP1, 3, 4, and 5 is associated with lung cancer (Hoque et al., 2006; Machida et al., 2011; Xie et al., 2012). Similarly, increased level of AQP5 has been reported in chronic myelogenous leukemia (Chae et al., 2008), esophageal cancer (Liu et al., 2013), and pancreatic cancer (Burghardt et al., 2003). During tumor proliferation, AQP5 interacts with the Ras-MAPK pathway and cyclin D1/CDK4 complexes in colon cancer and with the EGFR/ERK1/2/p38 MAPK signaling cascade in lung cancer, resulting in enhanced proliferation, differentiation and survival (Kang et al., 2008; Zhang et al., 2010).

### AQPs in Cerebral Edema and Ischemic Stroke

Cerebral edema plays a central role in the pathophysiology of many neurological disorders, including ischemic injury, traumatic brain injury, brain tumors leading to elevated intracranial pressure, decreased cerebral blood flow, ischemia, cerebral herniation, and death (Zador et al., 2009; Filippidis et al., 2016). AQP4 expressed in perivascular astrocyte end-feet plays a homeostatic role in water exchange between brain parenchyma and CSF in the ventricular and subarachnoid compartments (Amiry-Moghaddam et al., 2003). In the ischemic stroke mouse model, the presence of AQP4 was shown to aggravate post-ischemic cytotoxic edema as measured by post-ischemic hemispheric enlargement, while AQP4-null mice showed an opposite effect with an improved neurological outcome (Manley et al., 2000; Papadopoulos and Verkman, 2007). In the acute water intoxication model, AQP4-knockout mice had markedly reduced mortality from hyponatremia compared to WT mice. The protection attributed by the absence of AQP4 may be linked to reduced blood-brain barrier water permeability and a reduced rate of water flux into the brain parenchyma (Papadopoulos and Verkman, 2007). As a bidirectional water channel, AQP4 facilitates brain water accumulation in cytotoxic edema and clearance of excess brain water in vasogenic and interstitial edema (Verkman, 2012). In a mouse model of transient cerebral ischemia, AQP4 expression was rapidly up-regulated in perivascular end-feet, reaching the first peak after 1 hour (h) coinciding with early cerebral swelling in the core and border of the lesion, and the second peak in the penumbra after 48 h correlating with the degree of brain swelling (Ribeiro Mde et al., 2006). This finding suggests that AQP4 could be the major water channel involved in water movements after transient cerebral ischemia.

### AQPs in Renal Diseases

In the kidney, eight AQPs are expressed in different segments and various cells to maintain normal urine concentration and renal function (Noda et al., 2010; Li et al., 2017). Among them, AQP2 that is crucial for urine concentration function is regulated by transcriptional factors and post-transcriptional modifications for its expression and function (He and Yang, 2019). Mutation or functional loss of AQP2 leads to NDI, a rare disease characterized by polyuria and polydipsia (Kotnik et al., 2007). The significant up-regulation of AQP5 in the kidney tissue of diabetic nephropathy (DN) patients leads to polyuria (Afkarian et al., 2016).

Chronic kidney disease (CKD) is a multifactorial disorder playing an important role in diabetes, hypertension, dyslipidemia and proteinuria (Tsai et al., 2016). A cohort study among 620 patients with CKD revealed that AQP11 rs2276415 variant is associated with CKD progression (Han et al., 2019). AQP1 is expressed in the epithelia lining 71% renal cysts in human autosomal dominant polycystic kidney disease (ADPKD), 44% of which are derived from the proximal tubules (Bachinsky et al., 1995). Two-thirds of the cysts express either AQP1 or renal collecting duct water channel AQP2 (Devuyst, 1998). It is reported that the overexpression of AQP1 inhibited renal cyst development by restraining Wnt/β-catenin signaling in an orthologous ADPKD mice model (Wang W. et al., 2015). On the other hand, the deletion of *AQP1* promoted cyst development in embryonic kidney with polycystic kidney diseases (PKD) in mice. Analysis of the renal phenotype of AQP11-null mice showed that their kidneys are large, anemic and polycystic, which is similar with PKD in human. The cysts are absent in the medulla but abundant in the cortex where AQP11 is highly expressed (Li et al., 2017). The expression of AQP6 becomes changed with the development of renal cell carcinoma and oncocytoma (Yusenko et al., 2009; Tan et al., 2010).

### AQPs in Obesity

Obesity is defined as the enlargement and inflammation of adipose tissues, which is one of the most important metabolic disorders of this century and its deposition in internal organs is a major risk for the development of diabetes, dyslipidemia, hypertension, atherosclerosis, cardiovascular, and neurodegenerative diseases commonly recognized as metabolic syndrome (Zimmet et al., 2001; Flegal et al., 2012). AQPs play an important role in adipose tissue biology as well as the onset of obesity. AQP7, an aquaglyceroporin that can release glycerol from adipocytes to tissue interstitium (Hara-Chikuma et al., 2005), has been identified in human and mice adipose tissues (Kuriyama et al., 1997; Ishibashi et al., 1998; Rojek et al., 2008) and adipocytes (Kishida et al., 2000; Miranda et al., 2010). Higher expression of AQP7 was observed in obese insulin-resistant mice and adipose tissues of type 2 diabetic rats compared to their control groups, suggesting that dysregulation of AQP7 might be associated with increased input of glycerol for hepatic gluconeogenesis and increased glucose level in type 2 diabetes (Kishida et al., 2000; Lee et al., 2005; Rojek et al., 2008). AQP7-null mice showed accumulation of cellular glycerol, triacylglycerols (TAG) and glycerol kinase up-regulation, which leads to development of progressive adipocyte hypertrophy and early obesity onset (Hara-Chikuma et al., 2005; Hibuse et al., 2005). However, although AQP7-null mice in some other studies did not confirm obesity development, all these studies confirmed the association of AQP7 with glycerol metabolism (Matsumura et al., 2007; Skowronski et al., 2007). The down- and up-regulation of *AQP7* gene by feeding and fasting concomitant with glycerol production from endogenous TAG and lipolysis, respectively, are inversely related with plasma insulin levels (Kishida et al., 2000; da Silva and Soveral, 2017). In obese individuals, down-regulation of AQP7 in subcutaneous fat is associated with fat accumulation and adipocyte hypertrophy, however, it’s up-regulation in visceral fat is correlated with lipolysis (Rodríguez et al., 2011; Madeira et al., 2015). However, AQP7-null mice still show measurable glycerol secretion, indicating the presence of other glycerol transporters rather than AQP7 (Kishida et al., 2000). Indeed, among the other aquaglyceroporins, AQP3, 9, and 10 are found in the PM of adipocytes in subcutaneous and visceral adipose tissue, and additionally AQP3 in intragranular membranes (da Silva and Soveral, 2017). However, further research is necessary to investigate whether they are associated with obesity or not.

### AQPs in Cataracts

Natural congenital autosomal dominant cataracts are clinically diverse and genetically heterogeneous due to AQP0 mutations in humans (Bateman et al., 2000; Berry et al., 2000; Geyer et al., 2006) and mice [Cataract Fraser (Cat^*Fr*^) (Shiels and Bassnett, 1996); cataract lens opacity (Cat^*lop*^) (Shiels and Bassnett, 1996); cataract Tohoku (Cat^*Tohm*^) (Okamura et al., 2003)]. Knockout of AQP0 in mouse also resulted in cataract (Shiels et al., 2001).

### AQPs in Skin Diseases

Skin AQPs aforementioned are expressed in deep to superficial including hypodermis, dermis and epidermis, and their mutation, dysregulation, malfunction or dysfunction lead numerous skin diseases (Patel et al., 2017). Although it is not yet confirmed, an inflammatory skin disease, erythema toxicum neonatorum characterized with papules or pustules only in infants is supposed to be associated with increased levels of AQP1 in epidermis, dermis, and blood vessels (Marchini et al., 2003; Patel et al., 2017). A recent study with keratinocyte cell lines has hypothesized that down-regulation of AQP1, 3, and 9 might be concomitant with the aging of the skin induced by long-term exposure to blue light (Avola et al., 2018).

Down-regulation and/or mislocalization of AQP3, the most abundant and largely studied aquaglyceroporin in the skin, are associated with psoriasis (Lee et al., 2012; Patel et al., 2017; Bollag et al., 2020) and vitiligo (Kim and Lee, 2010; Esmat et al., 2018). However, although some studies have reported increased mRNA levels of AQP3 in proliferating keratinocytes in psoriasis (Bowcock et al., 2001; Swindell et al., 2014), its expression is down-regulated with later differentiation of keratinocytes (Zheng and Bollinger Bollag, 2003). Another study reported that AQP3-null mice showed reduced psoriatic lesion development and epidermal hyperplasia, and suggested that AQP3 levels remained unchanged in psoriasis (Hara-Chikuma et al., 2015). Therefore, it is still controversial whether AQP3 is up- or down-regulated in psoriasis (Patel et al., 2017). Controversial observations are also reported on non-melanoma skin cancers (NMSC), basal and squamous cell carcinoma (SCC) involving AQP3. While a study with immunohistochemical approach revealed the down-regulation of AQP3 in NMSC and SCC (Seleit et al., 2015), another study observed up-regulation of this protein in both diseases (Ishimoto et al., 2012). The later observation was supported by the study showing AQP3-null mice resistant to tumor formation in the model of SCC development (Hara-Chikuma and Verkman, 2008). The up-regulation of AQP3 both in mRNA and protein levels has been demonstrated in atopic dermatitis lesions or eczema in human patients and mouse models (Olsson et al., 2006; Nakahigashi et al., 2011). The down-regulation of AQP3 is supposed to be associated with xeroderma observed in diabetes because streptozotocin-induced diabetic mice showed decreased levels of AQP3 with reduced dermal water content (Ikarashi et al., 2017; Bollag et al., 2020). Recent studies demonstrated that down-regulation of AQP3 might be involved in some other skin diseases such as bullous pemphigus (Korany et al., 2019) and symmetrical acrokeratoderma (Rong et al., 2019); however, it’s up-regulation might be involved in scleroderma/systemic sclerosis (Luo et al., 2016).

Although limited studies have been done on the association of other AQPs with skin disorder, in patients with palmoplantar keratoderma, a skin disease characterized by hyperkeratosis in the soles and palms, mutations in AQP5 have been identified (Blaydon et al., 2013; Cao X. et al., 2014; Krøigård et al., 2016). A study shows that dendritic cells deficient in AQP7 reduce their antigen presentation (Hara-Chikuma et al., 2012b). The pathophysiologic phenotypes in the skin due to loss of AQP9 and 10 or their malfunction or dysfunction are not yet known, it is supposed that these AQP homologs might have shared similar functions as AQP3 (Patel et al., 2017).

## Aquaporins as Potential Drug and Diagnostic Targets

Aquaporins that facilitate the transport of water, the essential molecule of life, and some other physiologically important small molecules, and even ions, are expressed in all the organs of the human body (Figure 1 and Table 1). Discussion aforementioned reveals that the dysregulation, mutation, dysfunction and malfunction of AQPs have been associated as a key event with different life-threatening infectious and non-infectious diseases. Consequently, it has been essential to modulate the function or expression of AQPs during numerous infectious and non-infectious pathologies including different types of cancers, edema, obesity, brain injury, glaucoma, type 2 diabetes, NDI, AKI, CDK, DN, skin diseases, sepsis and sepsis-associated cholestasis, endotoximia and endotoximia-induced AKI, infective endocarditis, diarrhea, NMOSD, malaria, schistosomiasis, leishmaniasis, and several other conditions. Therefore, AQPs have been explored as an important drug and diagnostic target. Numerous studies have proposed many AQP-specific modulators and/or inhibitors including heavy metal-based inhibitors, cysteine inhibitors, small molecule inhibitors to modulate or inhibit the function and expression of human AQPs and parasite AQPs (Fadiel et al., 2009; Verkman et al., 2014; Wang J. et al., 2015; Esteva-Font et al., 2016; Méndez-Giménez et al., 2018; Abir-Awan et al., 2019; Hara-Chikuma et al., 2020).

Mercury inhibits the water transport function in AQPs with Cys 189, such as AQP1 (Esteva-Font et al., 2016). Similarly, heavy metal-based AQPs inhibitors such as silver- and gold-containing compounds were investigated as potential anticancer and anti-diabetic drugs in animal or human cell lines (Delporte et al., 2009; Madeira et al., 2014; Méndez-Giménez et al., 2018). Mercury and these heavy metals-based compounds interact with the cysteine in the vicinity of the highly conserved NPA motifs and inhibit the AQP permeability by causing blockage of the pore or conformational changes of the channel (Preston et al., 1993; Niemietz and Tyerman, 2002; Martins et al., 2012). However, mercury and heavy metals-based compounds cause irreversible cytotoxicity in human cells, and therefore, small molecule inhibitors/modulators without heavy metals to inhibit/modulate the function and expression of AQPs may have clinical implications against several diseases including cancers, diabetes and others (Best et al., 2009; Wang J. et al., 2015; Méndez-Giménez et al., 2018; Abir-Awan et al., 2019). The currently available AQP inhibitors, their structures, and the AQPs inhibited by them are listed in a very recent study (Abir-Awan et al., 2019). Acetazolamide, carbonic anhydrase inhibitor, used in glaucoma to reduce aqueous humor production, have been shown as irreversible inhibitor of AQP1 and 4 which protected tumor from cytotoxic edema and promoted tumor metastasis in glioma (Gao et al., 2006; Wang J. et al., 2015). Tetraethylammonium inhibits human AQP1, 2 and 4 expressed in *Xenopus* oocytes (Abir-Awan et al., 2019). Besides, anti-epileptic drugs such as topiramate, zonisamide, and lamotrigine are thought as possible inhibitors of AQP1, 4, and 5 although there is no conclusive evidence as safe AQP inhibitors (Shank and Maryanoff, 2008; Yang et al., 2008). AQP4-target inhibitor TGN-020 and aquaglyceroporins AQP3, AQP7 and AQP9-targeted inhibitors phloretin and compounds DFP00173 and Z433927330 have been identified (Abir-Awan et al., 2019; Sonntag et al., 2019). Auphen, an inhibitor of AQP3 that localize in the PVM between the host cell and the *Plasmodium* parasite reduces *P. vivax* liver hypnozoite and schizont burden, and inhibits *P. vivax* asexual blood-stage growth, and thus suggest that the AQP3 may be targeted for malaria treatment (Posfai et al., 2020). However, while some molecules are inhibitory in one study have no activity in other study due to variability within the experimental methods (Abir-Awan et al., 2019). Moreover, targeting the mechanism of calmodulin-mediated cell-surface localization of AQP4 instead of directly targeting the channel activity offers a new alternative smart approach for anti-edematous therapy (Kitchen et al., 2020).

It is certainly stimulating that the *AQP* gene (AQP1-cDNA) transfer has been developed and is under clinical trial (Baum et al., 2012). However, more studies are necessary to verify the efficacy and safety of *AQP* gene transfer. The recent discovery of new monoclonal antibody prevented macrophage-dependent liver injury by inhibition of AQP3-mediated H_2_O_2_ transport (Hara-Chikuma et al., 2020). Although the anti-AQP4 IgG poses no effect on water transport, it is under clinical trials for neuromyelitis optica treatments (Levy, 2017). However, AQP4-IgG possess diagnostics values in patients with NMOSD (Holmøy et al., 2020). In DN, AQP2 and 5 in urine can be used as potential novel biomarkers with sensitivity and early appearance (Wu et al., 2013; Lu et al., 2016; Han et al., 2019). Furthermore, AQP6 has been proposed as a biomarker for renal cancer diagnosis (Yusenko et al., 2009; Tan et al., 2010). In addition, AQP3, 5, 8, and 9 might be potential biomarker for several cancers of the digestive system namely colorectal, gastric, esophageal, and hepatocellular cancers (Nagaraju et al., 2016). However, there are still a lot of challenges in the way to develop AQPs-target modulators. Therefore, more studies with optimum high-throughput analysis are necessary to identify AQP modulators which are clinically urgent. Furthermore, AQPs as the potential diagnostic targets deserve additional studies.

Again, while *Plasmodium* spp. *Toxoplasma gondii* express one and two AQP homologs, respectively, *Trypanosoma cruzi* and *Leishmania* spp. encode up to five AQPs (Von Bülow and Beitz, 2015). In parasites, these AQPs are involved in multiple physiological processes such as regulation of osmotic pressure, nutrient uptake, and metabolic product efflux and/or host-parasite interactions (Von Bülow and Beitz, 2015; Ni et al., 2017; Ma et al., 2019). Furthermore, AQPs are the most abundant proteins in some parasites (Castro-Borges et al., 2011). Consequently, AQPs in parasites have become an important drug target (Ni et al., 2017). Although limited studies have been done on drug-like inhibitors for apicomplexan AQPs (Song et al., 2012; Meier et al., 2018), PbAQP-null *P. berhei* showed conspicuously reduced growth, virulence and progression through the liver stage (Promeneur et al., 2007; Promeneur et al., 2018). The TbAQP2 of three AQPs from *Trypanosoma brucei* plays an important role to uptake pantamidine, an anti-trypanosomal drug (Uzcategui et al., 2004; Von Bülow and Beitz, 2015; Song et al., 2016). AQPs in *Leishmania major* (LmAQP1) and *Schistosoma mansoni* (SmAQP) serve as channels for metalloids such as As and Sb from the anti-parasitic drugs (Gourbal et al., 2004; Faghiri and Skelly, 2009; Mukhopadhyay and Beitz, 2010; Mukhopadhyay et al., 2011). Furthermore, the SmAQP facilitates the transport of glycolytic-end product lactate to escape from toxicity (Faghiri et al., 2010; Meier et al., 2018). Substitution of Arg in the selectivity filter of LmAQP1 to Ala or Lys minimized the transport of As and Sb in cells, and thus increased the resistance to As and Sb in comparison with cells expressing the WT LmAQP1 (Figarella et al., 2007). Another study shows that the side chain of Ala163 in LmAQP1 may play role in drug resistance due to steric hindrance effect, and substitution of Thr164 to Cys imparts the mercury sensitivity by blocking the channel by HgCl_2_ (Mukhopadhyay et al., 2011). Very recently, it has been shown that RNA silencing treatment significantly reduced mRNA of TcAQP1 in adult *Toxocara canis*, a neglected parasitic nematode, keeping the phenotypic characteristics unchanged (Ma et al., 2019). The TcAQP1 has been suggested as a channel for drug uptake as the TcAQP1-knockout *T. canis* compromised the nematocidal activity of albendazole *in vitro*. These studies collectively suggest that parasite AQPs might be important candidate for therapeutic targets and drug entry routes. However, with human AQPs, researchers have to find out the specific effectors of pathogen AQPs with high affinity considering large number of studies. The physiological role of AQP has been extensively investigated only in *E. coli* and in lesser extent in *Pseudomonas aeruginosa*, *Streptococcus mutans*, and *Lactobacillus plantarum* (Tong et al., 2019). Therefore, to target the bacterial AQPs, the physiological role of this protein in bacteria should be established because the genomes of all bacteria have no *AQP* gene (Tong et al., 2019).

## Conclusion and Future Perspectives

Humans and pathogens always struggle each other to survive, and for this, both parties use a wide range of different strategies and learn from the counterpart to evolve in close connection. The host–pathogen interaction is a dynamic, ever evolving process. Intriguingly, increasing studies aforementioned support the notion that AQPs are important players in the host–pathogen interaction. This review will further increase the understanding about the involvement of AQPs in infectious and non-infectious diseases. Several physiologically relevant substrates get access to cells via AQPs that can largely influence the cellular behavior, which will ultimately determine either the disease development or cell defense mechanisms. Furthermore, AQPs are the key players for maintenance cellular and tissue homeostasis during inflammation, the common event in disease development. Dysregulation, dysfunction and malfunction of AQPs during the disease development are therefore assuming an increasing translational value in pathophysiology with promising medical applications. The regulatory mechanisms of AQPs in infectious and non-infectious diseases seem to be tissue- and AQP-specific (Tables 1, 2 and Figure 1). Therefore, intensive research should be focused on the functional regulation of AQPs during infectious and non-infectious diseases. Investigation on the regulation and functional roles of AQPs would not only provide novel insights on the diagnosis and prognosis of diseases, but also facilitate the development of potential therapeutics. Furthermore, intensive knowledge of AQP physiology would be one of the key ways to overcome the limitation of currently available AQP pharmacological modulators/inhibitors. The tissue-specific distribution of AQPs might suggest the necessity of tissue-specific or cell-specific discovery of AQP modulators. However, although the involvement of AQP in different life-threatening diseases including infectious diseases is quite clear, research on AQP-specific modulators are not advanced enough. The translation of AQP research into the drug development would open a new window to treat the life-threatening diseases. For translating the AQP research to solve the real life problems, researchers have to focus the future research for better understanding the mechanistic relationship between modulation of AQP function and a reduction in a specific disease. Additionally, targeting a fundamental cellular process such as altering the subcellular localization of an AQP, rather than trying to block the substrate accessible pore, would be a broadly applicable strategy for future AQP-based drug development (Kitchen et al., 2020). Besides, their broad range of substrate transport capacity, their additional involvement in interactome, signaling and trafficking indicates that researchers need more research for better understanding of AQP mystery. Furthermore, it is a challenge for researchers to establish the speculation of gases and ions permeation through the central pore of the AQP tetramer in addition to the physiological roles of these substances in the human body.

A mentionable limitation of this review is that several research outcomes were discussed from model animal studies for mimicking human physiology due to small numbers of studies with humans and human cells. Elucidation of mechanistic expression and functional regulation of AQPs in human studies might be helpful for developing tissue- and AQP-specific novel therapeutics.

## Author Contributions

AA and JA: concept and design. AA, TR, JA, AH, TE, and PC: partial manuscript writing. TR, JA, AH, and AA: figures and tables preparation. AA: data interpretation, compilation, supervision, and editing of the whole manuscript. All authors: contributed to the article and approved the submitted version.

## Conflict of Interest

The authors declare that the research was conducted in the absence of any commercial or financial relationships that could be construed as a potential conflict of interest.
